# Prevention and control of infectious disease transmission in subways: an improved susceptible–exposed–infected–recovered model

**DOI:** 10.3389/fpubh.2024.1454450

**Published:** 2024-12-12

**Authors:** Fang Zhou, Fang Hou, Jiangtao Wang, Qiaoyun Ma, Lanfen Luo

**Affiliations:** College of Information and Management Science, Henan Agricultural University, Zhengzhou, China

**Keywords:** SEIA, infectious disease transmission, subway protection measures, asymptomatic patient, infectious prevention and control

## Abstract

**Introduction:**

A well-connected transportation network unites localities but also accelerates the transmission of infectious diseases. Subways—an important aspect of daily travel in big cities—are high-risk sites for the transmission of urban epidemics. Intensive research examining the transmission mechanisms of infectious diseases in subways is necessary to ascertain the risk of disease transmission encountered by commuters.

**Methods:**

In this study, we improve the susceptible–exposed–infected–recovered (SEIR) model and propose the susceptible–exposed–infected–asymptomatic infected (SEIA) model. First, we added asymptomatic patients to the improved model as a parameter to explore the role of asymptomatic patients in the transmission of infectious diseases in a subway. The numbers of boarding and alighting passengers were added to the model as two time-varying parameters to simulate the exchange of passengers at each station.

**Results:**

The improved model could simulate the transmission of infectious diseases in subways and identify the key factors of transmission. We then produced an example of the transmission of coronavirus disease (COVID-19) in a subway using real subway passenger data substituted into the model for the calculations.

**Discussion:**

We ascertained that the number of exposed people continuously increased with the operation of the subway. Asymptomatic patients had a greater impact on the transmission of infectious diseases than infected people in the course of transmission. The SEIA model constructed in this study accurately determined the spread of infectious diseases in a subway and may also be applicable to studies on the transmission of infectious diseases in other urban public transport systems.

## 1 Introduction

Infectious diseases pose a long-term threat to human health and disrupt the normal social order. Infectious diseases are continuous and fast-spreading diseases that can be transmitted by an infected person to more than one person, exponentially increasing the total infected population. Common infectious diseases include swine influenza, avian influenza in birds, severe acute respiratory syndrome (SARS), coronavirus disease (COVID-19), dengue fever, and malaria. People are at risk of exposure to viruses and diseases that can affect their normal lives during daily commuting or by participating in social activities. The spread of infectious diseases has accelerated because of population growth and improved transportation systems.

COVID-19 aroused extensive worldwide attention on infectious diseases during the related global pandemic. COVID-19 was rapidly dispersed internationally because of its wide distribution and difficulties with protection. Research into the impact of transportation on the spread of epidemics had increased during the SARS period (1). Some scholars observed that the contact rate was a key parameter in the study of the evolution of diseases (2). After the outbreak of COVID-19 in Wuhan, many scholars paid close attention to the pandemic (3) and conducted research using mathematical models (4, 5). To predict the trend of a pandemic slowdown, there are articles which studied the outbreak of COVID-19 in Greece using a time series model, probability distribution, and a susceptible–infected–recovered (SIR) model (6). Some researchers noted that the COVID-19 pandemic could spread in family settings (7). Among the first scholars to study the spread of COVID-19 on buses, Edwards et al. (8) confirmed the effectiveness of surgical masks and the use of air conditioning systems to suppress the spread of the virus. Moghadas et al. (9) believed that the vast majority of COVID-19 incidences were related to a silent transmission caused by a combination of pre-symptomatic diagnosed patients and asymptomatic infected patients.

The susceptible–exposed–infected–recovered (SEIR) model is suitable for the study of transmission trends because susceptible individuals do not always develop symptoms immediately after infection. Tang et al. (10) used the SEIR model to calculate and analyze data during the outbreak of the pandemic in Wuhan and explored the implementation effects of various intervention measures. Xue et al. (11) observed that the Omicron variant of COVID-19 was more infectious than the Delta variant and seasonal influenza; however, its mortality rate was lower. The high infectivity of the Omicron variant has ensured the continuation of COVID-19 infections, increasing the risk of infection among people. Prem et al. (12) used an age-structure-based SEIR model and observed that measures used to maintain a physical distance had different implementation effects in different age groups. Maintaining a certain social distance effectively reduced the incidence rate of infections in school-age children and the older adult.

Transportation as a requisite for daily commuting should not only provide travel but also prevent the large-scale spread of epidemics (13, 14). It is imperative to adopt effective epidemic prevention measures in transportation. There are researchers who have found epidemic prevention measures such as city closures and travel restrictions on domestic airlines were effective (15), based on passenger volume data from Japan's public transportation network. Anderson et al. (5, 16) affirmed the contribution of vaccine developments, patient isolation, and self-protection to suppress the spread of epidemics. Some researchers posited that the channels connecting an epidemic area to other areas should be controlled during the early stages of an epidemic (17). When an epidemic spreads, relevant departments can effectively prevent the further spread of infections such as COVID-19 through the control of transportation hubs. Liu et al. (18) observed that the spread of an epidemic could effectively be prevented by implementing traceability measures to promptly isolate infected individuals and their close contacts. Rail networks are an important aspect of an urban public transportation system; they are also a critical component in epidemic prevention measures (19). Research into the transmission of asymptomatic infections in urban rail networks has received little attention in consideration of epidemic prevention measures. In this study, we included asymptomatic infected patients and explored the spread of infectious diseases and related factors within the subway system according to subway passenger flow.

Beginning in 1927, the SIR model (20) marked the inception of mathematical modeling within the field of epidemiology. Since that time, a variety of mathematical models built upon this foundational framework have been continuously developed and extensively discussed. During the SARS epidemic, many researchers used the SEIR model developed by the SRI model as the basis to further explore and change the mathematical model to deal with the problems at that time (21, 22). In the COVID-19 era, the SEIR model is still a common tool for many scholars to find ways to prevent the epidemic in the face of a more complex environment. Several studies have used SEIR models to talk about the changing patterns of the global pandemic (23, 24) or to predict the effectiveness of government anti-epidemic policies (25, 26). In this study, taking the subway in Z city as an example, the SEIR model was further innovated, and a model that can be used to simulate the spread of COVID-19 in the subway was proposed.

We constructed an improved SEIR model called the SEIA (susceptible–exposed–infected–asymptomatic infected). The SEIA model considered asymptomatic infected patients to be virus spreaders. We studied the transmission mechanism of infectious diseases in subway systems with highly concentrated populations, based on the impact of changes in passenger flow and infection rates on the spread of infectious diseases. The key elements influencing the spread of infectious diseases in the subway were analyzed in the model calculations on the basis of the scale of exposed people. This enabled us to understand the spread of infectious diseases in a subway and further analyze and predict trends in the spread of diseases. Our research extends and complements prior theoretical research on the spread of infectious diseases in urban rail systems.

## 2 Theory

### 2.1 SIR model

**Figure 1:**
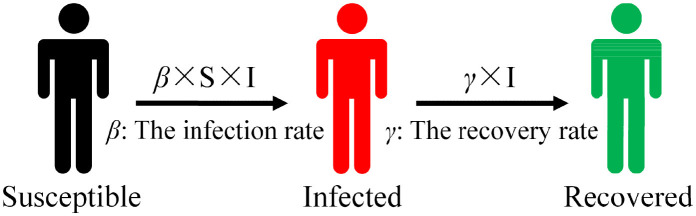
Susceptible–infected–recovered (SIR) model.

The mathematical models of infectious diseases define the categories of different populations based on their different states and then divide them into a square or rectangular warehouse. Figure 1 depicts a warehouse model in which susceptible (S) is classified as a susceptible warehouse, infected (I) is classified as an infected warehouse, etc. The state of different types of people changes in a warehouse model, so researchers can reassign people to corresponding warehouses according to their new transformed state. If a susceptible (S) individual is infected, that individual is transferred to the infected warehouse. After treatment and recovery, infected (I) individuals enter the recovered warehouse. This model is usually represented by differential equations that can be used to predict the number of infected individuals, the scale of infections, and the duration of the epidemic.

Common warehouse models include SI, SIR, and SEIR. The SIR warehouse model (20) (as illustrated in Figure 1) is a relatively basic infectious disease model that is suitable for the study of diseases such as smallpox and parotitis, which occur quickly but produce antibodies after recovery to ensure no immediate re-infection. The initial total population is assumed in the model without considering the migration status of the population and increases or decreases in birth and death rates. The number of infected (I) individuals increases by $\beta \times \frac{S}{N}\times I$ within a certain period of time, and the total number of people is *N* = *S*+*I*. Recovered (R) comprises people who contain antibodies in their body after rehabilitation and who will not immediately be re-infected, so this number is not included in the total number of people. Susceptible (S) individuals are transformed into infected (I) individuals with a probability of β after contact with infected (I) individuals in the SIR model. Assuming that the recovery rate of infected (I) individuals from the state of illness to the state of recovery is γ, then infected (I) individuals are cured with a probability of γ after a period of treatment to become a recovered (R) individual. The specific formula for the SIR model is as follows:


(1) $$\{\begin{array}{c} \frac{dS}{dt}=-\beta \frac{S\times I}{N}\\ \frac{dI}{dt}=\beta \frac{S\times I}{N}-\gamma I\\ \frac{dR}{dt}=\gamma I \end{array}\;and\;N=S+I$$

During the transmission process, infected (I) individuals have the ability to transmit disease after being infected and can spread to *R*_0_ individuals on average during the period of disease (*R*_0_ = β*/*γ in the absence of any intervention measures). When *R*_0_ > 1, the number of infected (I) individuals monotonically increases toward the highest value; when *R*_0_ < 1, the number of infected (I) individuals monotonically decreases, leading to the final elimination of the disease.

### 2.2 SEIR model

**Figure 2:**
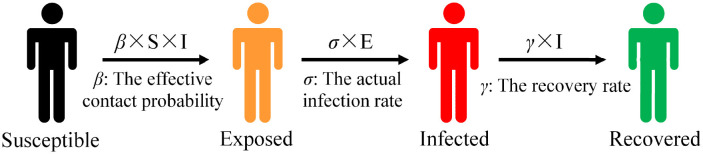
Susceptible–exposed–infected–recovered (SEIR) model.

Certain diseases such as COVID-19 have an incubation period. After a susceptible (S) individual encounters an infected (I) individual, the susceptible person is not immediately infected with the disease; a period of incubation is required to develop the disease. This group is known as exposed (E). Figure 2 presents the state transition diagram of the SEIR model.

Exposed (E) individuals transform into infected (I) individuals based on infection rate of σ. The infection rate σ is usually the inverse of the average incubation period. The differential equation of the SEIR model is represented as follows:


(2) $$\{\begin{array}{c} \frac{dS}{dt}=\;-\beta \frac{S\times I}{N}\\ \frac{dI}{dt}=\;\beta \frac{S\times I}{N}-\sigma E\\ \frac{dI}{dt}=\;\sigma E-\gamma I\\ \frac{dR}{dt}=\;\gamma I \end{array}\;,\;and\;N=S+E+I$$

Susceptible (S) individuals are transformed into exposed (E) individuals with a probability of β after contact with infected (I) individuals in the SEIR model. There is an infection rate of σ in a population of exposed (E) individuals that causes exposed (E) individuals to be infected with the disease. Thus, infected exposed (E) individuals move from the exposed warehouse to the infected warehouse. Patients in the infected warehouse are cured based on a probability of γ after treatment and become recovered (R) individuals. Consequently, they move from the infected warehouse and enter the recovered warehouse.

We primarily considered infectious diseases with latent periods that result in the creation of antibodies within a short period of recovery such as H1N1 and COVID-19. The SEIR model provides a greater alignment with research requirements than infectious disease models such as SI and SIR because of the addition of the exposed (E) and recovered (R) categories of population segmentation. The basic assumptions of classical infectious disease models do not consider factors such as population migration and natural death, so they are suitable only for the study of the short-term process of virus transmission in subway carriages. We chose the SEIR model as the basic model to study the transmission mechanism of infectious diseases in subways.

### 2.3 SEIA model

#### 2.3.1 Model assumptions

The traditional SEIR model involves four types of people: susceptible (S), exposed (E), infected (I), and recovered (R). Infected (I) individuals may not experience a secondary transmission in a single ride because passengers may have a limited time traveling on a subway and there is a certain incubation period for exposed (E) individuals to transform into infected (I) individuals. The transmission model considers only the process of susceptible (S) individuals becoming exposed (E) individuals after they encounter infected (I) individuals.

Susceptible (S) individuals may not actually be infected after coming into contact with infected (I) individuals. In this study, this latent population was categorized as exposed (E). The asymptomatic infected (A) parameter was added to the model to construct the improved SEIR model because asymptomatic infected (A) individuals also have the ability to spread infections. Our model included the following four population types: susceptible (S), exposed (E), infected (I), and asymptomatic infected (A), abbreviated as SEIA. According to the particularities of the subway environment, *u*_*t*_was introduced as the number of people boarding at time *t* (a certain stop) and *g*_*t*_was used to depict the number of people alighting at time *t* (a certain stop). We studied the spread of infectious diseases in subways using these two parameters to simulate the increase or decrease in the number of people in the subway when a train stops.

In the formula, *S*_*t*_ is the number of susceptible (S) individuals in the subway at time *t*. *E*_*t*_ is the number of exposed (E) individuals in the subway at time *t*. *I*_*t*_ is the number of infected (I) individuals in the subway at time *t*. *A*_*t*_ is the number of asymptomatic infected (A) individuals in the subway at time *t*. *r* is the effective number of infected (I) and asymptomatic infected (A) individuals who encounter susceptible (S) individuals (the average number of carriers). β_1_ is the probability of susceptible (S) individuals being infected after contact with infected (I) individuals. β_2_ is the probability of susceptible (S) individuals being infected after contact with asymptomatic infected (A) individuals. ∑*g*_*t*_ is the total number of people alighting at all stops.

#### 2.3.2 Model establishment

The specific formula of the subway infectious disease transmission model is as follows:


(3) $$\{\begin{array}{c} \frac{dS_{t}}{dt}=-\frac{r\times s_{t}\left(\beta_{1}I_{t}+\beta_{2}A_{t}\right)}{N_{t}}+u_{t}-g_{t}\\ \frac{dE_{t}}{dt}=\frac{r\times s_{t}\left(\beta_{1}I_{t}+\beta_{2}A_{t}\right)}{N_{t}}\\ \frac{dI_{t}}{dt}=-\frac{g_{t}}{\sum g_{t}}\times I_{t}\\ \frac{dA_{t}}{dt}=-\;\frac{g_{t}}{\sum g_{t}}\times A_{t} \end{array}\;,N=S+E+I+A$$

The improved SEIR model added asymptomatic infected (A) individuals to the traditional model as the source of infection. This ensured its suitability for infectious diseases with a silent transmission such as the influenza of a virus and COVID-19. We assumed that the initial total population was *N* without considering the migration status of the population and increases or decreases in births and deaths. The formula for the increase in the number of exposed (E) individuals over a period of time is as follows:


(4) $$\begin{array}{l} \frac{r\times s_{t}\left(\beta_{1}I_{t}+\beta_{2}A_{t}\right)}{N_{t}} \end{array}$$

The total number of people in the SEIA model was calculated as *N* = *S*+*E*+*I*+*A*. Susceptible (S) individuals were transformed into exposed (E) individuals after contact with infected (I) individuals or asymptomatic infected (A) individuals. The number of susceptible (S) individuals changed in different ranges because trains constantly stop at stations and passengers constantly enter and leave subways. Correspondingly, the number of exposed (E) individuals increased as the number of subway stops increased. The number of exposed (E) individuals reached maximum value when the train arrived at the final stop.

**Figure 3:**
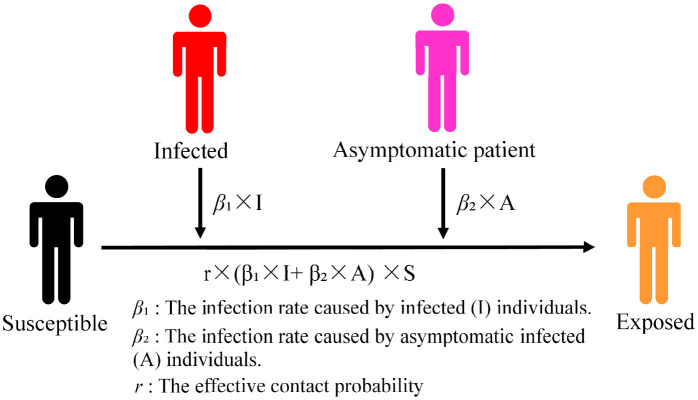
Schematic diagram of subway infectious disease transmission model.

The model diagram of the improved SEIR model is depicted in Figure 3.

Not all susceptible (S) individuals are directly exposed to infection within the contact range of infected (I) or asymptomatic infected (A) individuals. This may expose certain susceptible (S) individuals to the range of the virus transmission. It is not guaranteed that susceptible (S) individuals exposed within the range of virus transmission will contract the virus; rather, they have an infection probability of β. Susceptible (S) individuals who encounter infected (I) individuals may be infected with a virus at an infection rate of *r* × β_1_. Susceptible (S) individuals who encounter asymptomatic infected (A) individuals may be infected with the virus at an infection rate of *r* × β_2_ and can transform into exposed (E) individuals. A certain period of incubation is required before determining whether individuals have been infected with a disease and for symptoms to appear. The exposed (E) category only indicates the population that may be infected; it is not equivalent to those infected during a single ride or encounter with a subway.

#### 2.3.3 Propagation process

Certain susceptible (S) individuals transform into exposed (E) individuals after contact with infected (I) or asymptomatic infected (A) individuals with the probability of *r* × β_1_ or *r* × β_2_. With each subway or stop, *u*_*t*_is added to the number of susceptible (S) individuals and *g*_*t*_is deducted from the number of susceptible (S) individuals. Infected (I) and asymptomatic infected (A) individuals decrease in proportion to $\frac{g_{t}}{\sum g_{t}}$ with each stop. The process of virus transmission begins from the time infected (I) and asymptomatic infected (A) individuals enter the subway and ends when there are no infected (I) individuals in the subway. We used MATLAB to randomly iterate and generate the average passenger flow data of boarding and alighting.

A scenario analysis can be employed to study the spread of infectious diseases by assigning different values for the effective contact number *r*. The analysis can compare differences in the number of exposed (E) individuals with the use of protective measures inside the subway or not and can judge the effectiveness of the prevention of infection. The degree of transmission of different infection groups can be studied according to the different values of the infection rate of infected (I) and asymptomatic infected (A) individuals. Subsequently, the degree of influence of the two groups of infected (I) and asymptomatic infected (A) individuals on exposed (E) individuals can be ascertained. This enables the identification of key factors in the spread of infectious diseases in subways, the simulation of trends in the spread of infectious diseases, and the exploration of disease transmission patterns.

## 3 Case study

### 3.1 Background

**Figure 4:**
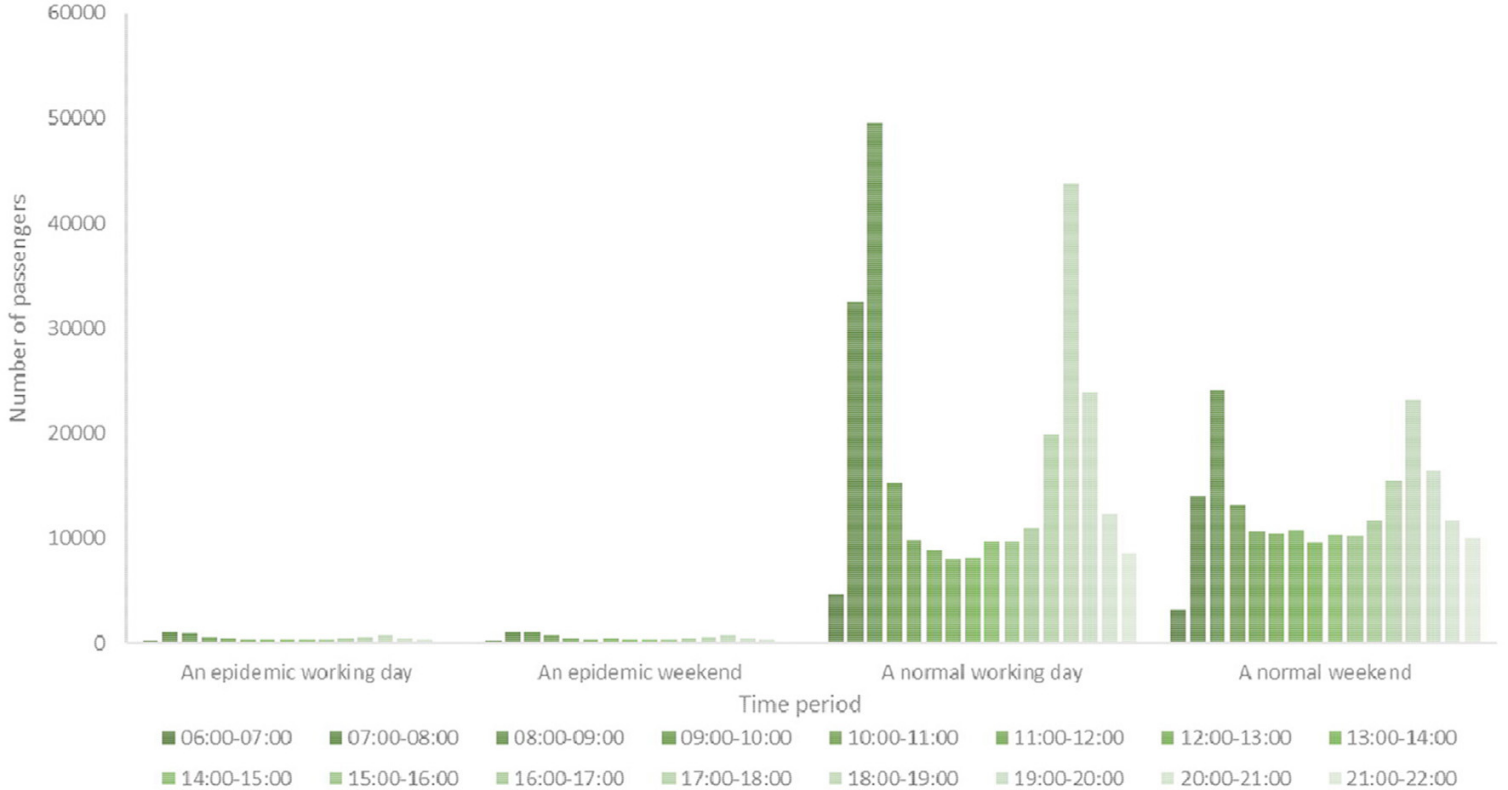
Comparing of passenger flow rates between normal daily and epidemic periods.

We used City Z in China as our case study for analysis. City Z has a population of over 10 million in the central region of China. City Z is a transportation hub with highways, railways, and aviation and information facilities. It has a transportation network composed of three modes of transportation: railways, highways, and aviation. An integrated urban public transportation system has also been formed within the city with rail and rapid transport as the backbone, conventional public transportation as the main body, and a slow traffic extension. Currently, there are seven rail transport systems in operation. We selected Line B as our research object because it was representative and had a large passenger flow. The comparison between the hourly passenger flow of Line B during daily periods and the hourly passenger flow during an epidemic period is illustrated in Figure 4.

Figure 4 illustrates that the hourly passenger flow of Line B in City Z significantly decreased to fewer than 2000 during an epidemic period of infectious diseases. However, the passenger flow Line B was >10,000 during a normal working day. It reached a peak passenger flow during peak weekdays, with a maximum of over 50,000 in Line B. The hourly passenger flow was relatively homogeneous at the weekend, but it still markedly increased during the peak period.

### 3.2 Without protective measures

We used the real passenger flow data of Line B subway in City Z as our original data. As not all newly infected (I) individuals use a subway on a particular day, the number of cases of infected (I) and asymptomatic infected (A) individuals on a certain day may be reduced. This reduced number was used in our model to simulate the spread of infected (I) individuals in the subway. In different scenarios, that is, where protective measures are and are not administered, a change in the number of exposed (E) individuals can reflect whether the protective measures are effective.

We were able to ascertain the effect of protective measures on the scale of exposed infections from the simulation results during the distribution of infectious diseases in a subway.

**Figure 5:**
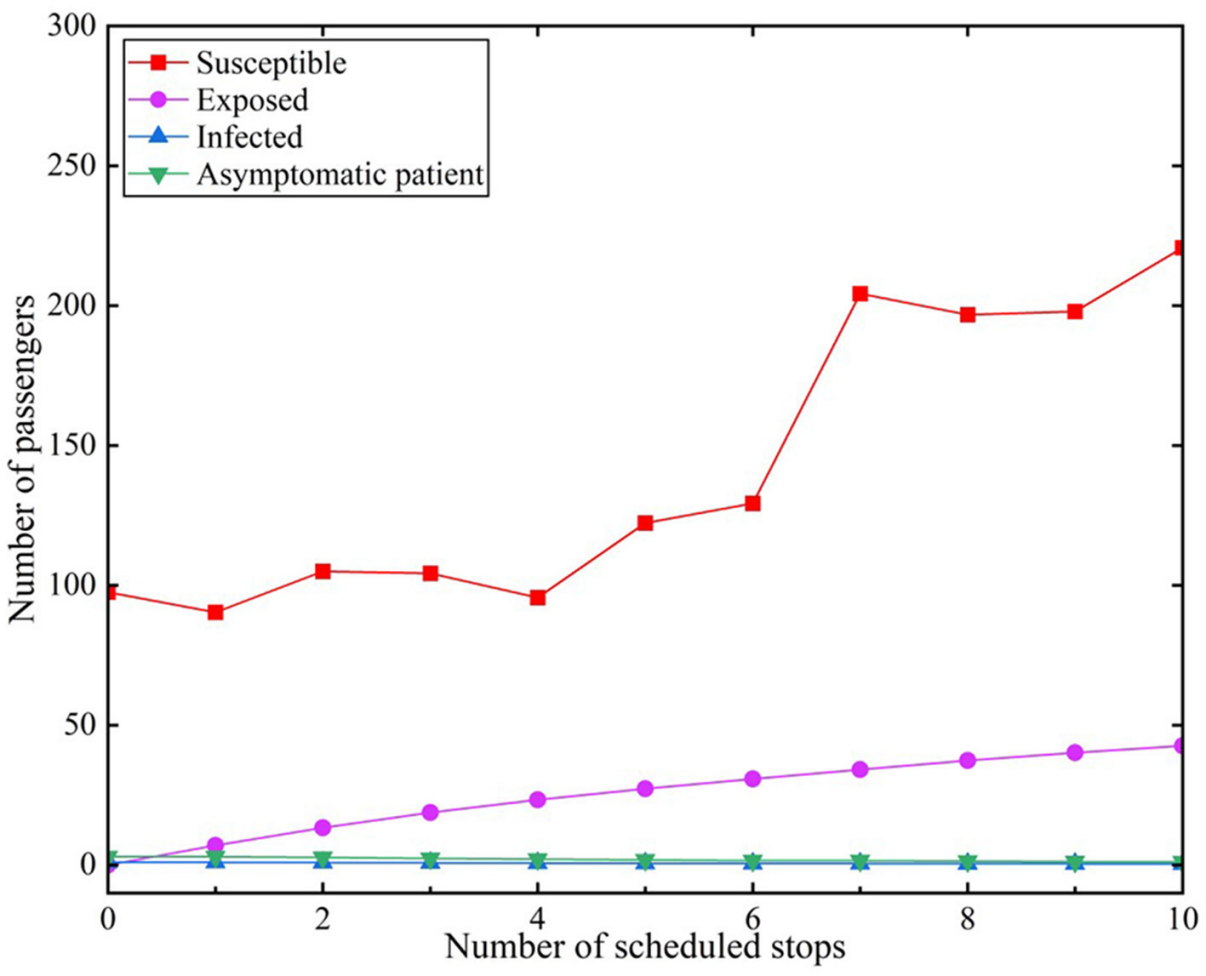
Number of exposed individuals without protective measures during peak hours.

We assumed that infected individuals encountered 10 stations accessed by subways during the peak period. The effective contact number *r* was set to 9.5 under the scenario of administering no measures (18). The infection rate of infected individuals was β_1_ = 0.2. Similarly, the infection rate of asymptomatic infected (A) individuals was β_2_ = 0.2. The results are depicted in Figure 5.

Figure 5 reveals that the number of susceptible (S) individuals in the subway gradually increased during the peak period with continuous stops in the subway system. The change in the number of susceptible individuals revealed a fluctuating upward trend, increasing from an initial 98 to 221 at the last stop. The curve of exposed (E) individuals gradually rose with an increase in stops, which indicated that the number of exposed individuals was related to the number of stops of the subway. There were 7.04 exposed individuals after the subway passed through one station; the number increased to 27.27 when it reached the fifth station. The number of susceptible dramatically changes in 6 to 7 stations. Subsequently, the rate of the increase in the number of exposed individuals becomes more gently until the tenth station, when it increased to 42.63.

The reason may be that there are many people boarding the train at the seven stations. We set the number of people who get on and off at each stop in the model, which is also a feature of our model. Our data are a random number that are randomly generated based on real statistics. The sixth station has a large number of people, and it may be that the sixth station is a larger station or transfer station. This phenomenon is also common in life.

### 3.3 With protective measures

**Figure 6:**
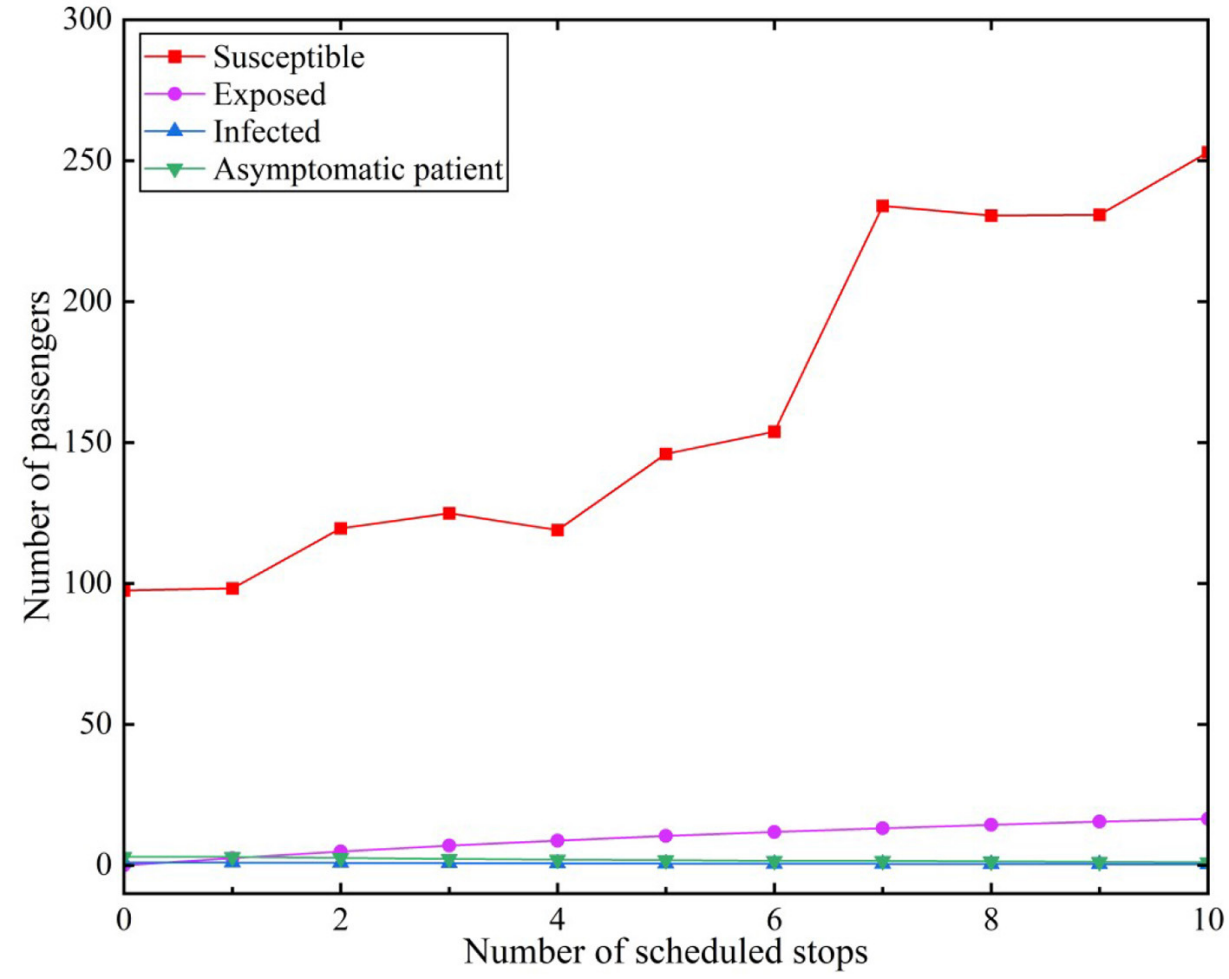
Number of exposed individuals with protective measures during peak hours.

We assumed that infected (I) individuals would use the subway during the peak period. The effective contact number *r* was set to 3.4 under the scenario of implementing preventative measures (18). The infection rate of infected individuals was β_1_ = 0.2. Similarly, the infection rate of asymptomatic infected (A) individuals was β_2_ = 0.2. The results are depicted in Figure 6.

Figure 6 reveals that the growth in the number of susceptible individuals presented a fluctuating upward trend. The number of susceptible individuals reached 253.02 at the tenth station. The increasing trend of the number of exposed individuals with protective measures tended to be more gently than with the situation without measures. The number of exposed individuals was only 2.58 when stopping at the first station, but it increased to 16.47 after passing ten stations.

It was evident that the increase in the number of susceptible individuals in the scenes with protective measures was greater than that without protective measures at each station when comparing the scenes with and without protective measures. The number of susceptible individuals slowly decreased after administering protective measures. The rate of transition from susceptible to infected individuals slowed. The number of exposed individuals decreased by 32.3 when preventative measures were implemented, presenting a reduction of 158.83% from the perspective of an increase or a decrease in the number of infected individuals and changes in their percentage. Thus, administering relevant protective measures is effective when using a subway. It is necessary to use certain protective measures in closed places such as subways, both for subway operations and personal travel.

## 4 Results and discussion

As a key parameter in the COVID-19 infection model, the infection rate β affected the number of exposed individuals. We analyzed the number of changes of infected and asymptomatic infected individuals under different infection rates by adjusting the transmission rate of symptomatic and asymptomatic infections β_1_ and β_2_. We determined the extents to which the two types of infected people had an influence on the trends in the spread of infectious diseases.

**Figure 7:**
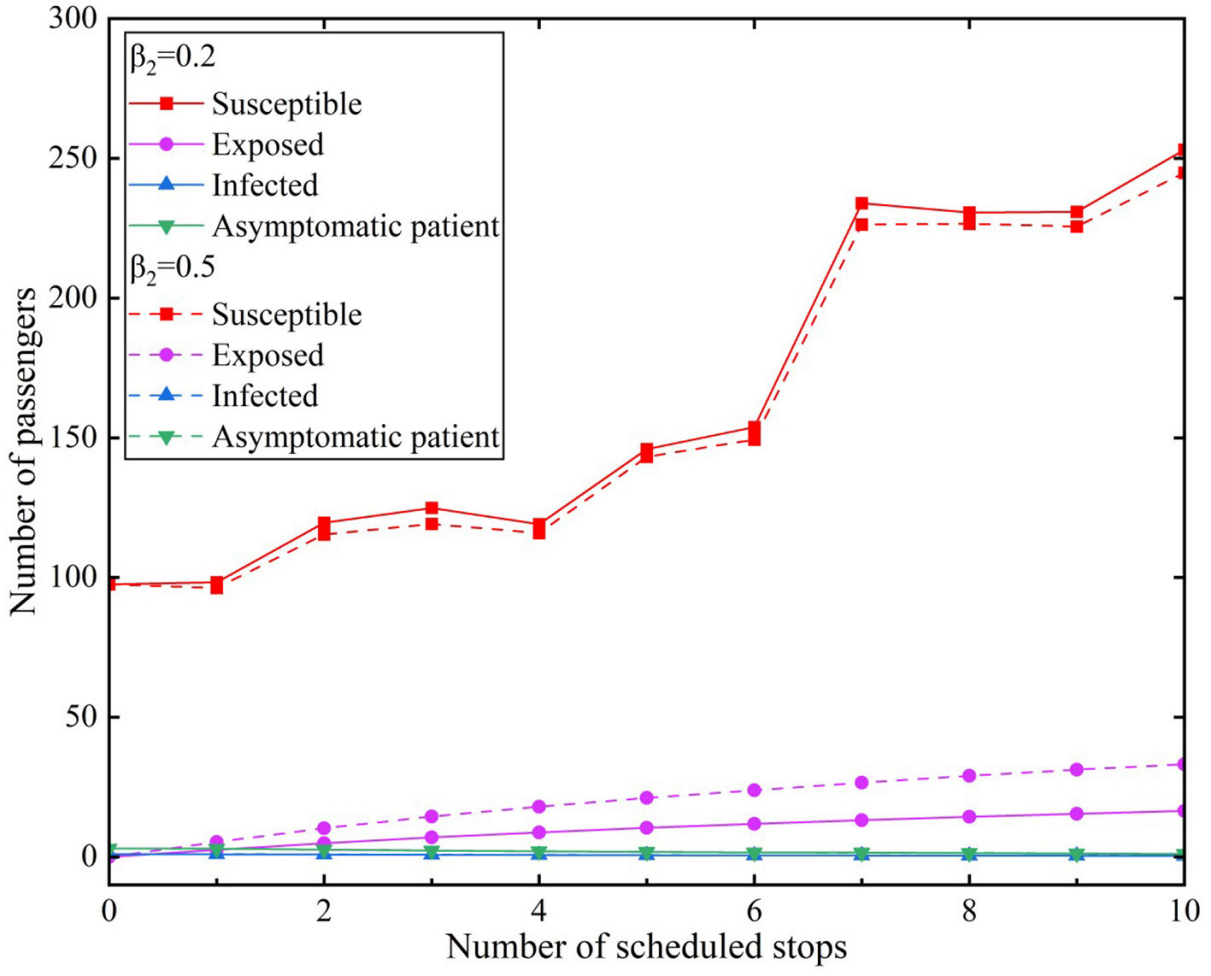
Simulation results of the model for peak hours under Scenario 1.

**Figure 8:**
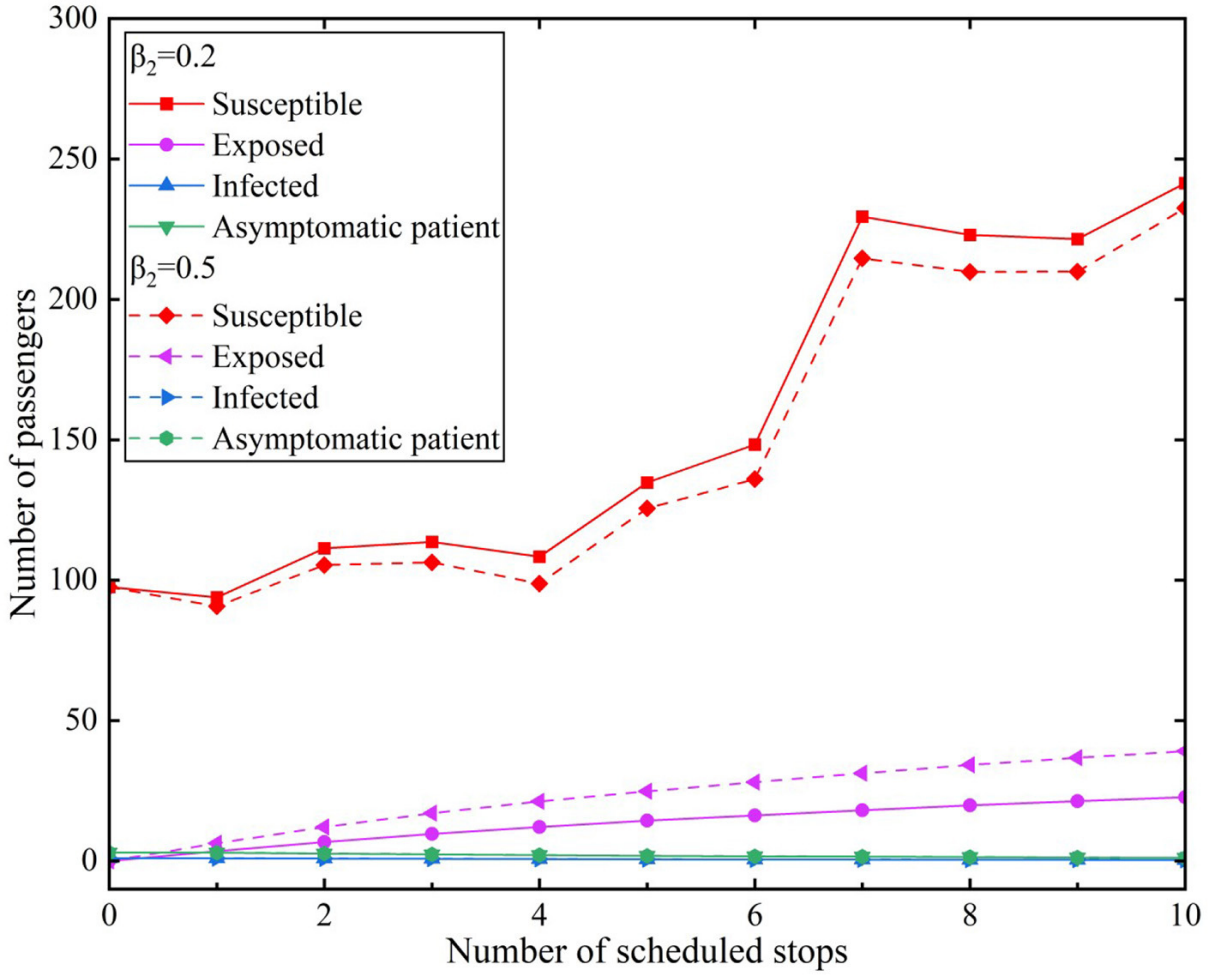
Simulation results of the model for peak hours under Scenario 2.

We used the average of the hourly passenger flow during a peak period of a certain day as the raw data and incorporated it into the propagation model. We assumed that infected individuals used the subway through 10 stations in the peak period for the convenience of comparison. The effective contact number was *r* = 3.4. We used two types of infection rates and compared them with two scenarios. The simulation results of the model experiments for the scenarios are presented in Figures 7, 8. Figure 7 illustrates Scenario 1, where β_1_ value was 0.2 and β_2_ values were 0.2 and 0.5, respectively. Figure 8 depicts Scenario 2, where β_1_ value was 0.5 and β*2* values were 0.2 and 0.5, respectively.

In Scenario 1, the transmission rate of infected individuals was β_1_= 0.2 and the β_2_infection rates of asymptomatic infected individuals were 0.2 and 0.5, respectively. The simulation results of different infection rates during the peak period are depicted in Figure 7.

The simulation results illustrated in Figure 7 revealed that the number of susceptible individuals during the peak period gradually decreased with an increase in the β_2_ values during the peak period. The number of susceptible individuals increased when the trains continued to stop at stations and new passengers entered the subway. The change in the number of the susceptible individuals was not significantly different, indicating that the results of different values in the scenario had little effect on susceptible individuals. The curve of exposed individuals revealed a steady upward trend that decreased after the seventh station; nonetheless, it continued to grow. The number of exposed individuals increased from the first station of 2.58 to 16.45 at the tenth station when the β_1_ value is 0.2. However, the number of exposed individuals increased from the first station of 5.36 to 33.13 at the tenth station when the β_1_ value is 0.5. According to the different values of β_1_, the number of exposed individuals has a difference of 16.68.

In Scenario 2, the transmission rate of infected individuals was β_1_= 0.5 and the β_2_infection rates of asymptomatic infected individuals were 0.2 and 0.5, respectively. The simulation results of different infection rates during the peak period are illustrated in Figure 8.

The number of susceptible individuals decreased with an increase in the β_2_ values in Scenario 2. The β_2_values for asymptomatic infected individuals were the highest because of the β_1_transmission rate of infected individuals. Thus, the corresponding curve of the number of exposed individuals was also the steepest in the two scenarios. The number of exposed individuals reached 22.69 at the tenth station of the subway when the β_2_ value is 0.2. Similarly, the number of exposed individuals reached 39.09 when the β_2_ value is 0.2. According to the different values of β_2_, the number of exposed individuals has a difference of 16.4.

We compared the different values of the β_1_ infection rate of infected individuals in the two scenarios. When the β_2_infection rate of infected individuals was 0.2, the numbers of exposed individuals were 16.45 and 22.69, respectively, according to the different values of β_1_. When the β_2_infection rate of infected individuals was 0.5, the numbers of exposed individuals were 33.13 and 39.09, respectively, according to the different values of β_1_. From a horizontal comparison of the β_2_ values in Scenarios 1 and 2, the number of infected individuals was >16. When comparing the β_1_infection rate of infected individuals with the β_2_infection rate of asymptomatic infected individuals, we observed that the β_2_values had a greater impact on exposed individuals.

Infected individuals may consciously avoid travel and self-test their health at home when they experience symptoms such as fevers and coughs. Infected individuals often choose self-driving, walking, or using well-ventilated public transportation when there is an essential requirement to travel. Certain passengers who use the subway may also consciously reduce their contact with other passengers during the subway ride. It is difficult for asymptomatic infected individuals to ascertain whether they are infected as they do not present clinical symptoms. Asymptomatic infected individuals may maintain normal social activities and may not consciously maintain a social distance or reduce activities in crowded places. The transmission caused by asymptomatic infected individuals in subways is more covert, causing difficulties for subways and leading to an accelerated spread of infectious diseases.

## 5 Conclusion

After a pandemic, the aim of prevention and control should shift to exploring trends in the spread of infectious diseases for daily epidemic prevention and control. The influence of different factors on the trend of an epidemic can be identified by exploring the mechanisms of the transmission of infectious diseases. Cost-effective dynamic prevention and control measures can be then administered based on these results.

The patterns of disease transmission must be studied to ascertain the transmission process of infectious diseases in subways. In this study, we first determined the number of effective contacts, the infection rate of infected individuals. We also added asymptomatic infected individuals to the SEIR model together with infected individuals as the source of infection in the transmission process of infectious diseases in subways.

We constructed an SEIA infectious disease transmission model based on the classic SEIR model. The SEIA model considered asymptomatic infected individuals and the uniqueness of subway operating sites. We added changes in the number of people boarding and alighting the subway to the process of the spread of an infection using subway passenger flow characteristics.

The model proposed in this study is suitable for the study of the spread of infectious diseases in subways. It could also be applied to other transportation systems. The accuracy of the model in future research could be improved by adding other factors such as the historical passenger flow of the route stations and if the stopping stations are in an epidemic area.

## Data Availability

The data analyzed in this study is subject to the following licenses/restrictions: the data presented in this study are available on request from the corresponding author. Requests to access these datasets should be directed to Fang Hou, hf_1778@163.com.

## References

[1] Chowdhury SMEK Forkan M Ahmed SF Agarwal P Ali ABMS Muyeen SM. Modeling the SARS-CoV-2 parallel transmission dynamics: asymptomatic and symptomatic pathways. Comput Biol Med. (2022) 143:105264. 10.1016/j.compbiomed.2022.105264 35182952 PMC8788092

[2] Goscé L Barton DAW Johansson A. Analytical modelling of the spread of disease in confined and crowded spaces. Sci Rep. (2014) 4:1–6. 10.1038/srep04856 24798322 PMC4010926

[3] Wei X Li M Pei X Liu Z Zhang J. Assessing the effectiveness of the intervention measures of COVID-19 in China based on dynamical method. Infect Dis Modell. (2023) 8:159–71. 10.1016/j.idm.2022.12.007 36624814 PMC9812467

[4] Rao IJ Vallon JJ Brandeau ML. Effectiveness of face masks in reducing the spread of COVID-19: a model-based analysis. Med Dec Making. (2021) 41:988–1003. 10.1177/0272989X211019029 34041970 PMC8484026

[5] Anderson RM Heesterbeek H Klinkenberg D Hollingsworth TD. How will country-based mitigation measures influence the course of the COVID-19 epidemic? Lancet. (2020) 395:931–4. 10.1016/S0140-6736(20)30567-5 32164834 PMC7158572

[6] Katris C. A time series-based statistical approach for outbreak spread forecasting: application of COVID-19 in Greece. Expert Syst Appl. (2021) 166:114077. 10.1016/j.eswa.2020.114077 33041528 PMC7531284

[7] Chan JF Yuan S Kok KH To KK Chu H Yang J. A familial cluster of pneumonia associated with the 2019 novel coronavirus indicating person-to-person transmission: a study of a family cluster. Lancet. (2020) 395:514–23. 10.1016/S0140-6736(20)30154-9 31986261 PMC7159286

[8] Edwards NJ Widrick R Wilmes J Breisch B Gerschefske M Sullivan J. Reducing COVID-19 airborne transmission risks on public transportation buses: An empirical study on aerosol dispersion and control. Aerosol Sci Technol. (2021) 55:1378–97. 10.1080/02786826.2021.1966376

[9] Moghadas SM Fitzpatrick MC Sah P Pandey A Shoukat A Singer BH. The implications of silent transmission for the control of COVID-19 outbreaks. Proc Nat Acad Sci. (2020) 117:17513–5. 10.1073/pnas.2008373117 32632012 PMC7395516

[10] Tang B Wang X Li Q Bragazzi NL Tang S Xiao Y. Estimation of the transmission risk of the 2019-nCoV and its implication for public health interventions. J Clin Med. (2020) 9:462. 10.3390/jcm9020462 32046137 PMC7074281

[11] Xue L Jing S Zhang K Milne R Wang H. Infectivity versus fatality of SARS-CoV-2 mutations and influenza. Int J Infect Dis. (2022) 121:195–202. 10.1016/j.ijid.2022.05.031 35584743 PMC9107628

[12] Prem K Liu Y Russell TW Kucharski AJ Eggo RM Davies N. The effect of control strategies to reduce social mixing on outcomes of the COVID-19 epidemic in Wuhan, China: a modelling study. Lancet Public Health. (2020) 5:e261–70. 10.1016/S2468-2667(20)30073-6 32220655 PMC7158905

[13] Xu XK Liu XF Wang L Wu Y Lu X Wang X. Assessing the spread risk of COVID-19 associated with multi-mode transportation networks in China. Fundam Res. (2023) 3:305–10. 10.1016/j.fmre.2022.04.006 PMC9023380 40477969

[14] Bian Z Zuo F Gao J Chen Y Venkata SSCP Bernardes SD. Time lag effects of COVID-19 policies on transportation systems: a comparative study of New York City and Seattle. Transpor Res Part A. (2021) 145:269–83. 10.1016/j.tra.2021.01.019 36569966 PMC9759401

[15] Murano Y Ueno R Shi S Kawashima T Tanoue Y Tanaka S. Impact of domestic travel restrictions on transmission of COVID-19 infection using public transportation network approach. Sci Rep. (2021) 11:3109. 10.1038/s41598-021-81806-3 33542248 PMC7862278

[16] Burki T K. Omicron variant and booster COVID-19 vaccines. Lancet Respir Med. (2022) 10:e17. 10.1016/S2213-2600(21)00559-2 34929158 PMC8683118

[17] Lu J Lin A Jiang C Zhang A Yang Z. Influence of transportation network on transmission heterogeneity of COVID-19 in China. Transpor Res Part C. (2021) 129:103231–103231. 10.1016/j.trc.2021.103231 34092940 PMC8169317

[18] Liu Y Rocklöv J. The effective reproductive number of the Omicron variant of SARS-CoV-2 is several times relative to Delta. J Travel Med. (2022) 29:taac037. 10.1093/jtm/taac037 35262737 PMC8992231

[19] Li X Zhou L Jia T Peng R Fu X Zou Y. Associating COVID-19 severity with urban factors: a case study of Wuhan. Int J Environ Res Public Health. (2020) 17:6712. 10.3390/ijerph17186712 32942626 PMC7558510

[20] Kermack WO McKendrick AG. A contribution to the mathematical theory of epidemics. In: Proceedings of the Royal Society of London Series A, Containing Papers of a Mathematical and Physical Character. (1927). p. 700–21. 10.1098/rspa.1927.0118

[21] Girardi P Gaetan C. An SEIR model with time-varying coefficients for analyzing the SARS-CoV-2 epidemic. Risk Analysis. (2023) 43:144–55. 10.1111/risa.13858 34799850 PMC9011870

[22] Ng T W Turinici G Danchin A. A double epidemic model for the SARS propagation. BMC Infect Dis. (2003) 3:1–16. 10.1186/1471-2334-3-19 12964944 PMC222908

[23] Li MY Graef JR Wang L Karsai J. Global dynamics of a SEIR model with varying total population size. Math Biosci. (1999) 160:191–213. 10.1016/S0025-5564(99)00030-9 10472754

[24] Li M Y Smith H L Wang L. Global dynamics of an SEIR epidemic model with vertical transmission. SIAM J Appl Math. (2001) 62:58–69. 10.1137/S003613999935986017443392

[25] Yang Z Zeng Z Wang K Wong S-S Liang W Zanin M. Modified SEIR and AI prediction of the epidemics trend of COVID-19 in China under public health interventions. J Thorac Dis. (2020) 12:165. 10.21037/jtd.2020.02.64 32274081 PMC7139011

[26] Efimov D Ushirobira R. On an interval prediction of COVID-19 development based on a SEIR epidemic model. Annu Rev Control. (2021) 51:477–87. 10.1016/j.arcontrol.2021.01.006 33623479 PMC7891093

